# Supplementary material for: Exceptional increase in the creep life of magnesium rare-earth alloys due to localized bond stiffening
Source: Nat Commun. 2017 Dec 8;8:2000. doi: 10.1038/s41467-017-02112-z (PMC5722870; doi:10.1038/s41467-017-02112-z)
Supplement: Supplementary file 1 — Supplementary Information [file 41467_2017_2112_MOESM1_ESM.pdf]

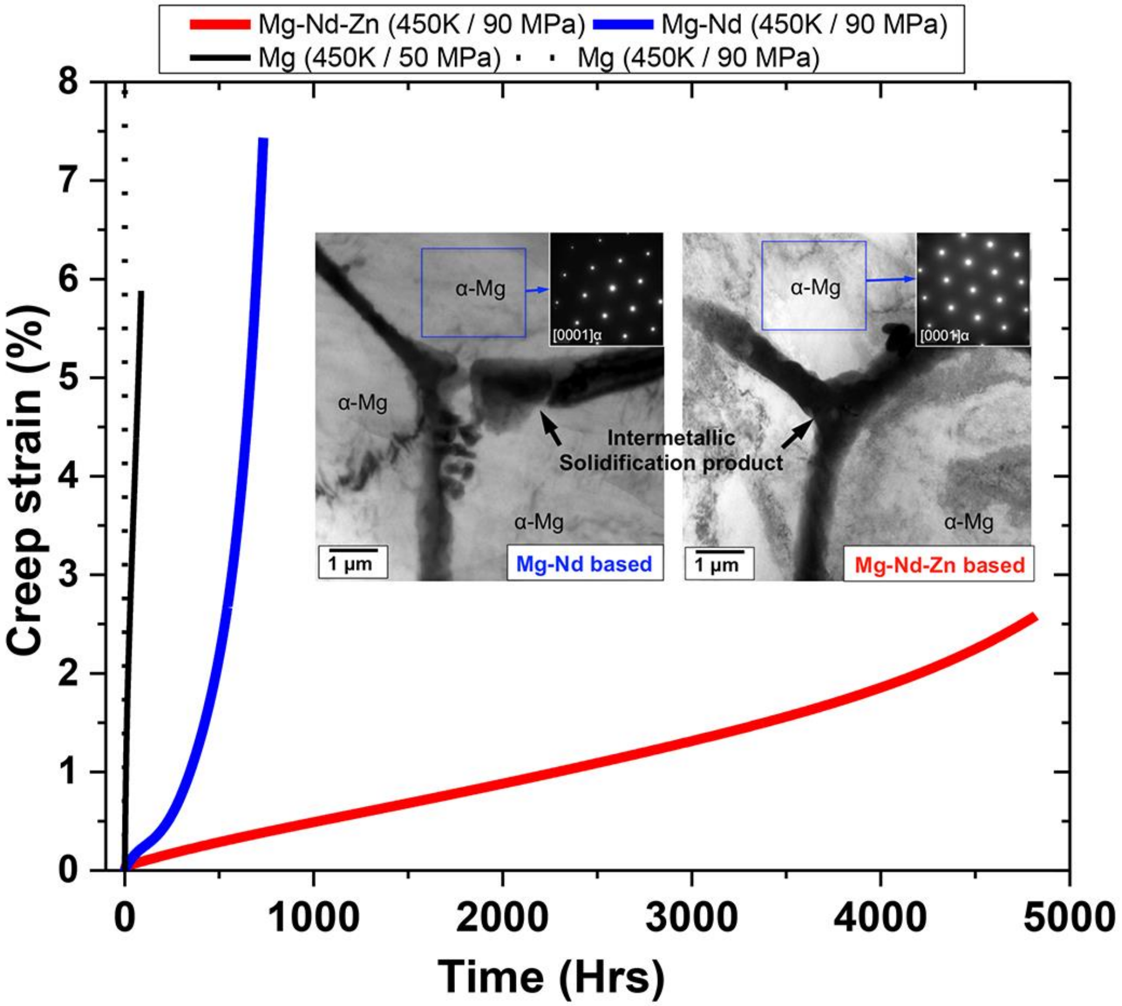

Supplementary Figure 1. Strain vs. time plots comparing the creep response of Mg-Nd and Mg-Nd-Zn alloys. Note that the creep response of Mg (solid and dotted black lines) is significantly different from the alloys. Inset shows the initial, as-solidified, microstructure consisting of large intermetallic solidification product and the primary α-Mg matrix. Selected area diffraction recorded along the [0001]α from the α-Mg matrix indicated no discernable extra precipitate reflections in both alloys.

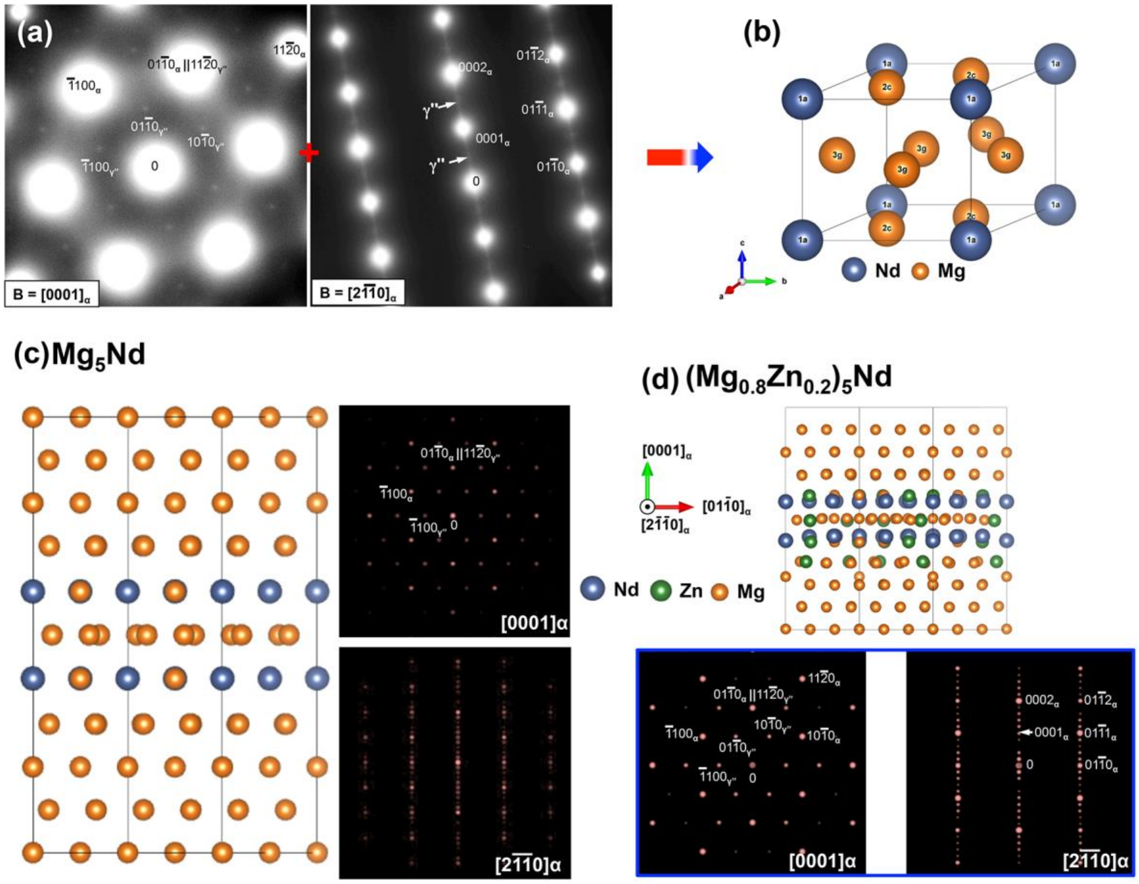

Supplementary Figure 2. (a) Selected area diffraction patterns (SADPs) along [0001]α and [2$\bar{1}\bar{1}$0]α zone axes reveal reflections from γ'' precipitates, (b) schematic crystal structure of pure Mg$_5$Nd γ''. (c)-(d) Simulated diffraction patterns from γ''–Mg structures obtained from DFT calculations shown: (c) Pure Mg$_5$Nd γ''–Mg and (d) (Mg$_{0.8}$Zn$_{0.2}$)$_5$Nd γ''–Mg. The simulated diffraction intensities from of γ''[(Mg$_{0.8}$Zn$_{0.2}$)$_5$Nd] – Mg is in excellent agreement with experimentally recorded the SADPs in (a). This demonstrates the validity of the chosen γ'' structures. In (c) and (d) depict the relaxed supercells after energy minimization.

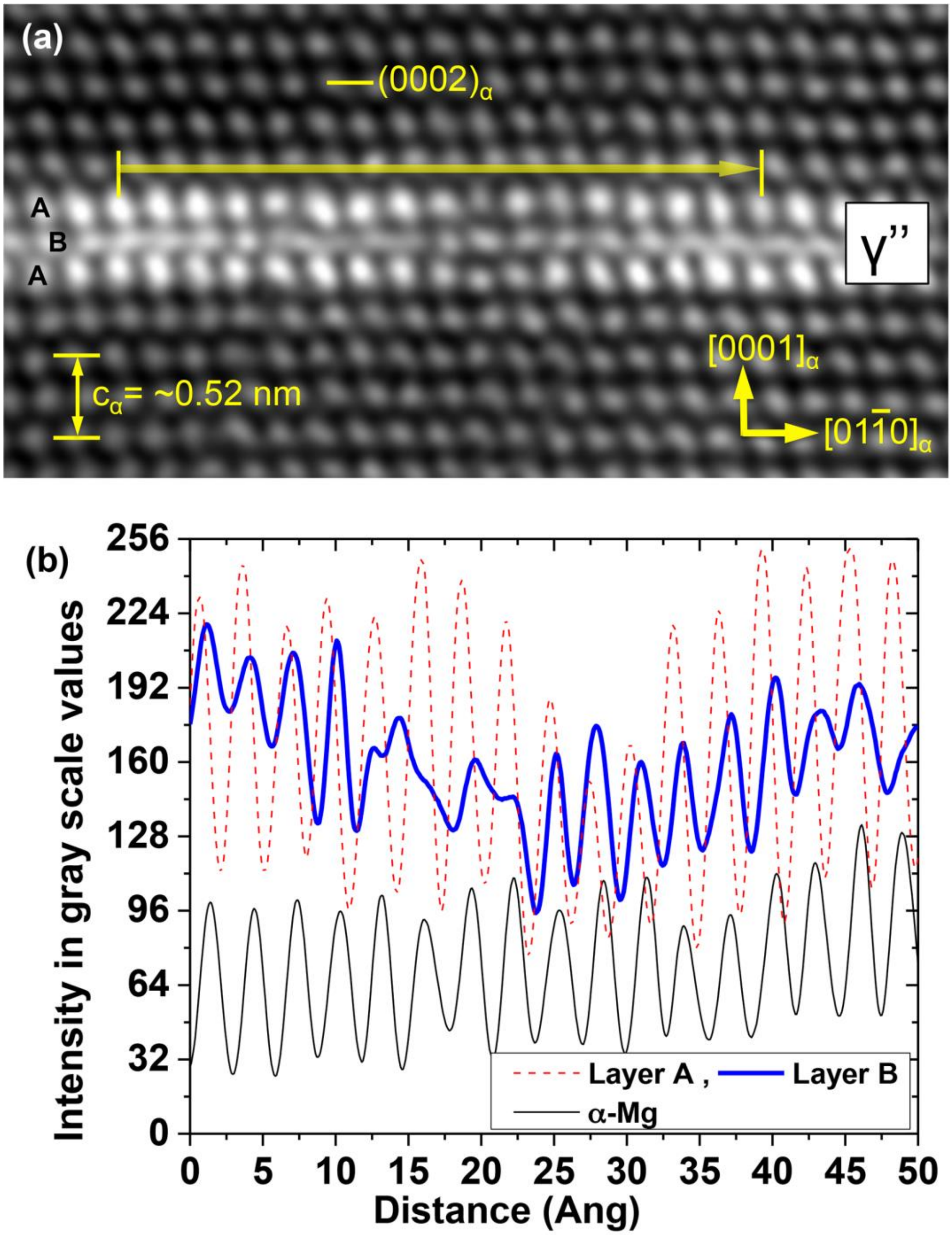

Supplementary Figure 3. (a) Aberration corrected HAADF-STEM atomic resolution image of the γ''–Mg interface. The $[2\bar{1}\bar{1}0]\alpha$ vector is perpendicular to the plane of (a). (b) Intensity profiles reveal intensities from γ'' are consistently higher than α-Mg; indicating the influence of heavier Nd and Zn within γ''.

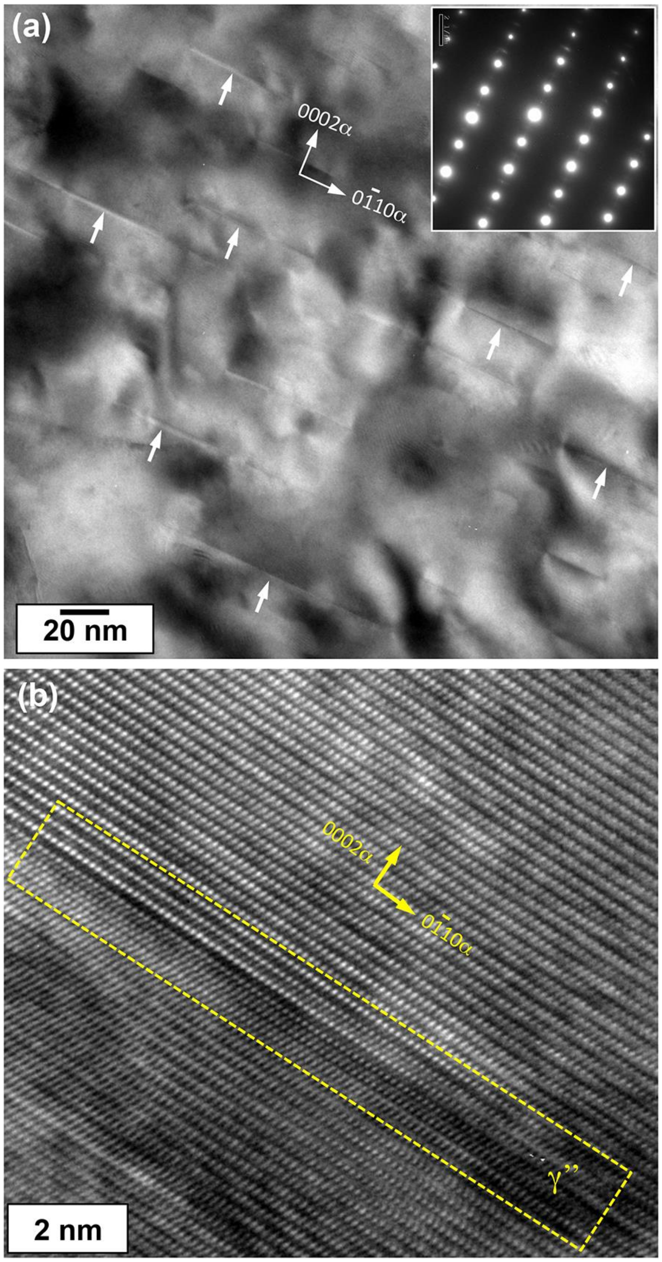

Supplementary Figure 4. Microstructure of Mg-Nd-Zn alloy after 4800Hrs of creep testing: (a) bright-field TEM image showing the existence of fine scale basal γ'' (indicated with arrows) and (b) High-resolution TEM recorded along $[11\bar{2}0]_{\alpha}$, from the region depicted with a dotted box shows that γ'' precipitates share a coherent interface with the parent Mg-matrix

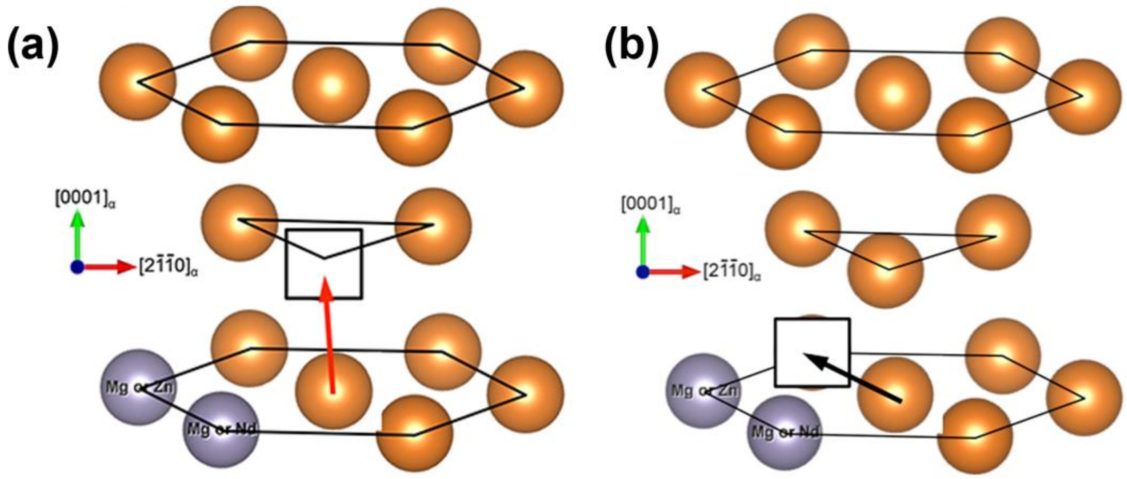

Supplementary Figure 5. Schematics diagram of vacancy, shown as a square, migration path during: (a) out-of-plane and (b) in-plane diffusion. The Nd and Zn atoms are colored purple.

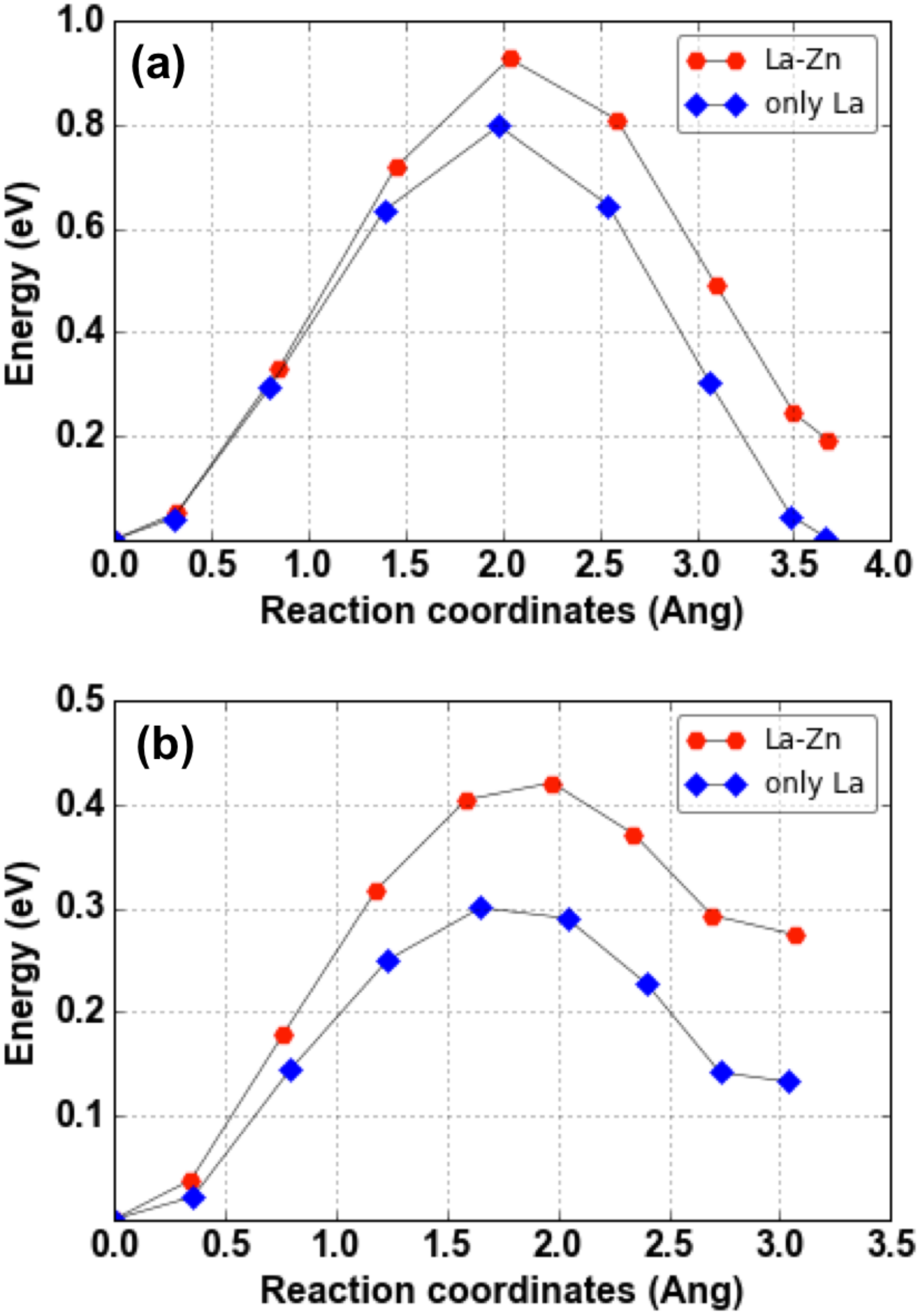

Supplementary Figure 6. Energy vs. reaction coordinates plots for (a) out-of-plane and (b) in-plane vacancy migration in Mg-La and Mg-La-Zn.

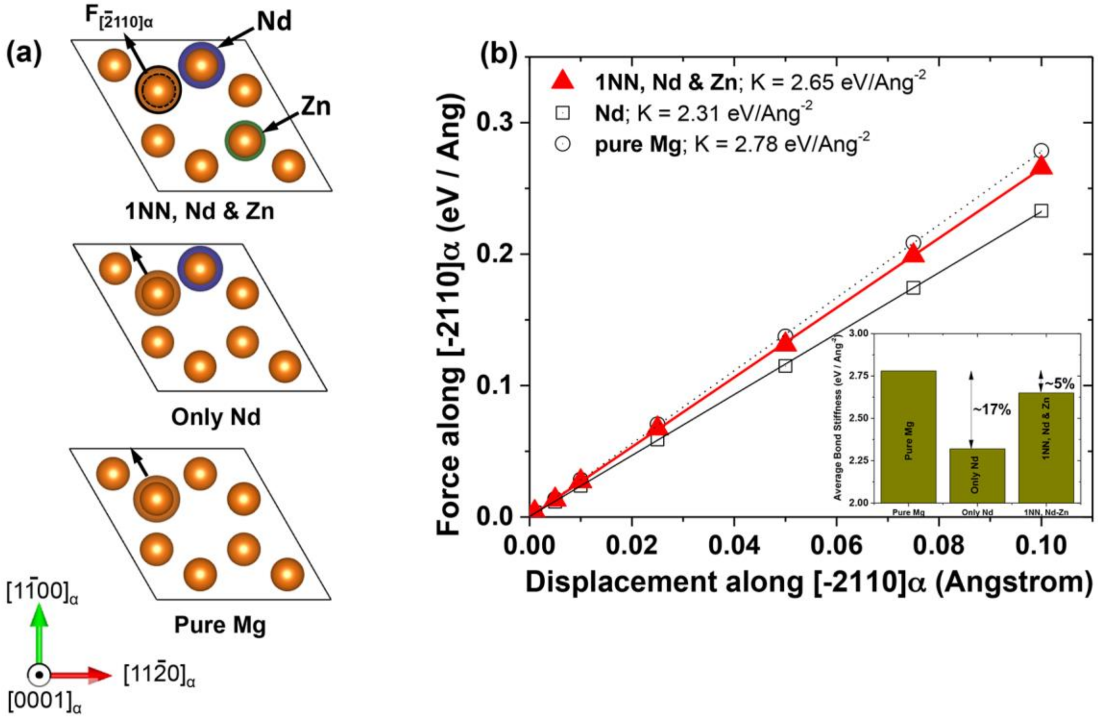

Supplementary Figure 7. (a) Schematics of supercells viewed along [0001]α. An Mg atom was displacement along [$\bar{2}$110]α in each supercell. (b) Force vs. displacement plots for Mg atom present in pure Mg, Mg-Nd and Mg-Nd-Zn. Note that, while the bond – stiffness, a measure of bond strength, for pure Mg and Mg-Nd-Zn are comparable, substitution of only Nd slightly reduces the bond strength. The bond strength along the a-axis follow the trend: Mg ≈ Mg-Nd-Zn > Mg-Nd. As a comparison, the bond strength along the c-axis is Mg-Nd-Zn > Mg-Nd > Mg.

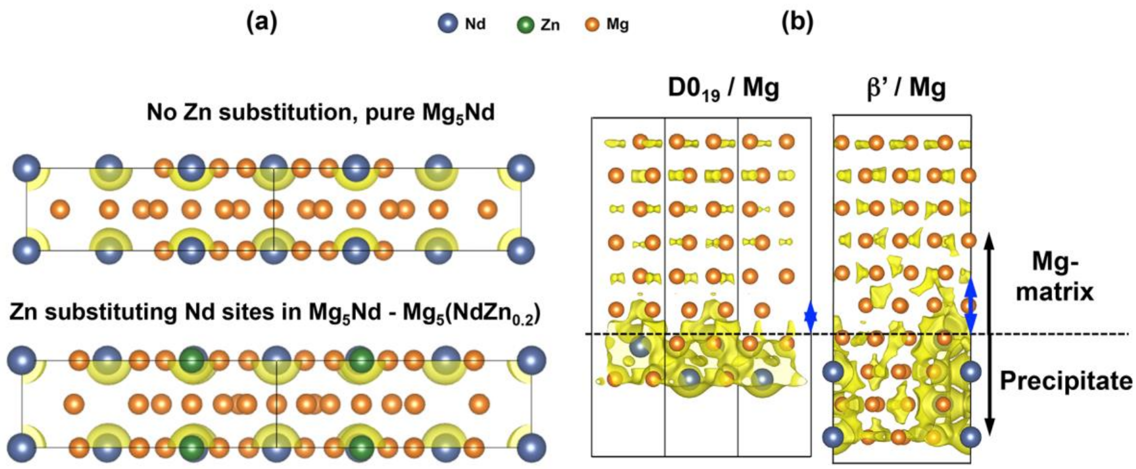

Supplementary Figure 8. Charge isosurfaces with $\Delta\rho = 0.015$ e$Å^{-3}$ showing (a) lack electronic charge delocalization between $(0001)_{\gamma''}$ planes in γ'' structures, and (b) partial charge delocalization along $[0001]_{\alpha}$ direction in DO_{19}–Mg and β'–Mg structures.

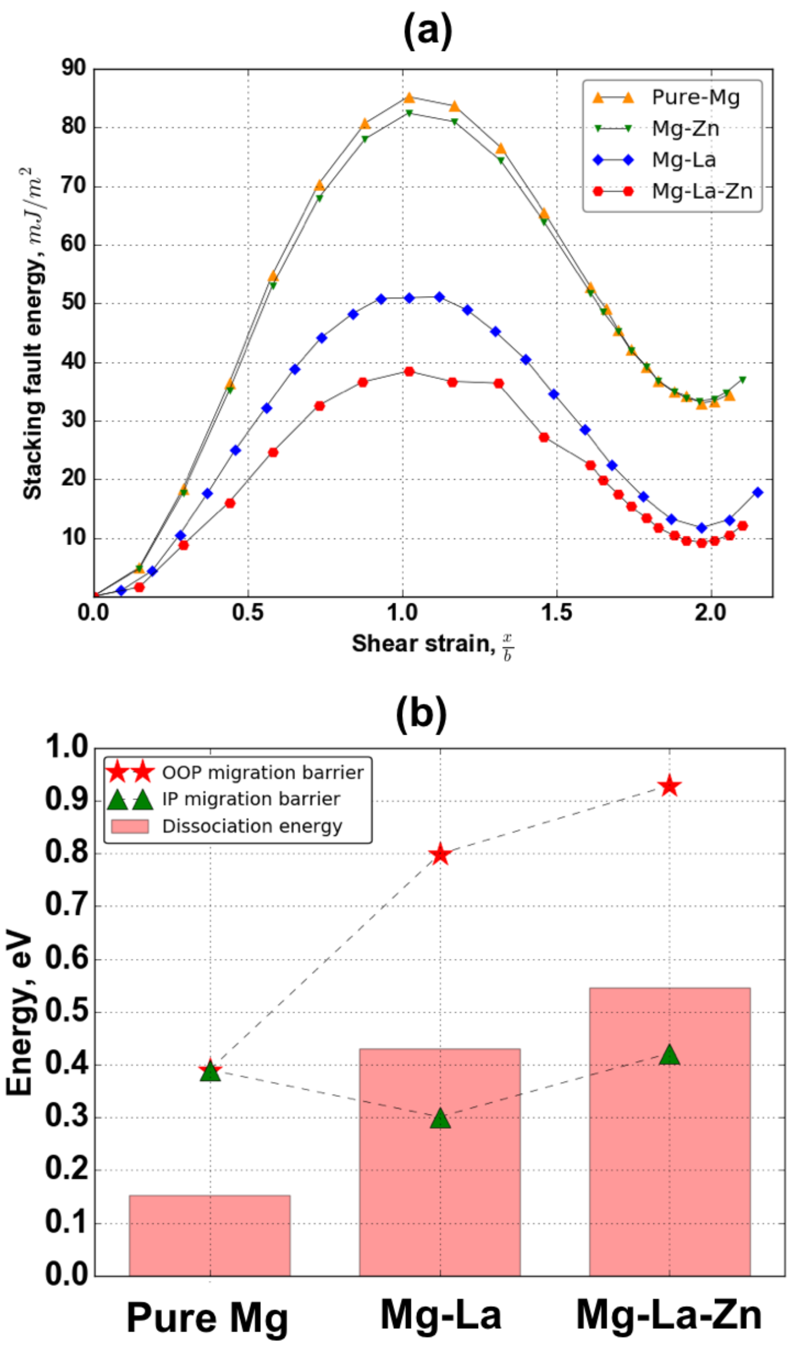

Supplementary Figure 9. (a) Plots comparing the generalized stacking fault energy (GSFE) curves of pure-Mg, Mg-Zn, Mg-La, and Mg-La-Zn. (b) Histogram comparing the energy required to dissociate a perfect (1/2)<11$\bar{2}$0>$_\alpha$ dislocation into two (1/3) <1100>$_\alpha$ partials in pure-Mg, Mg-La, and Mg-La-Zn are shown with in-plane and out-of-plane vacancy migration energies superimposed. The trends observed are consistent with Mg-Nd and Mg-Nd-Zn alloys.

Supplementary Methods

Experimental alloys and creep testing conditions and microstructural characterization. Two alloys of compositions Mg-0.6Nd-0.4La (in at.%) or Mg-Nd based, and Mg-0.6Nd-0.4La-0.3Zn or Mg-Nd-Zn based were prepared via high-pressure die-casting (HPDC). We also point out that La was added to each alloy in order to maximise the fluidity of the molten alloy, which eventually facilitates the die casting process [1]. Regardless, at the end of the supplementary section we have critically evaluated the role of La on creep deformation using published literature and our DFT calculations. Supplementary Table 1 gives the measured composition (in wt.%) of the two alloys, whilst Supplementary Table 2 lists the solubility of Nd, La and Zn in Mg.

The HPDC process involved preparing casting cylindrical dog-bone tensile bars (~5.6 mm gauge length) for both alloys with a 250 tonne Toshiba cold-chamber. The tensile bars were subjected to constant loads such that the tensile specimens nominally experience 90 MPa. Creep tests were carried out at 450 K, where the temperature control was better than ±1K. An extensometer attached to the specimen gauge was used to measure the strain in each alloy specimens during creep deformation at 450 K and 90 MPa.

Microstructures of Mg-Nd and Mg-Nd-Zn alloys before and after deformation was examined using transmission electron microscopy (TEM) in conventional and HAADF-STEM (high angle annular) modes, and 3D atom probe tomography (3DAP). TEM samples were prepared conventionally using 3 mm circular discs which were dimple ground (Fishione 300®) and ion milled (Gatan® 691) at 3.5 kV and 15 μA. The TEM foils were examined in FEI Tecnai G^2 TF20™ and FEI probe corrected Titan3™ 80-300 S/TEM. 3DAP experiments were carried out with a local electrode atom probe (LEAP 3000X HR®) from Cameca Inc. Conical shaped atom probe tips for 3DAP examination were extracted from the alloys via focused ion beam milling (FEI Nova NanoLab 200™). The tips were placed in the LEAP chamber in high vacuum (~10^{-10} bar) and maintained at 60 K. All atom probe experiments were conducted at in laser mode with a pulse rate of 160 kHz, pulse energy of 0.3 nJ at 30 K. Visualization and analysis of 3DAP results

were carried out Cameca's IVAS 3.6.6® (Integrated Visualization and Analysis Software). For very fine-scale precipitates e.g. γ'' (Figure 1(b)), whose composition is rather difficult to extract, we conducted cluster analyses utilizing the algorithms available in IVAS 3.6.6 ®. Briefly, in case of γ'' the algorithm identifies Nd and Zn ions at a critical distance from one another and labels them as clusters. Since the distance between the participating atoms are crucial, the algorithm statistically compares the identified clusters with a random solution (which is typically symmetrical Gaussian curve). For several datasets different values of critical distances were considered before the composition of the fine-scale precipitates was established [2,3]. We may point out that the limitations imposed by the detector efficiency (~37%) of our equipment (LEAP 3000X HR®), and fine scale of the precipitates, one can only report a range of compositions for these precipitates.

Supplementary Table 1. Compositions of Mg-Nd and Mg-Nd-Zn alloys measured with inductively coupled plasma atomic emission spectroscopy

Alloy	Nd wt%	La wt%	Zn wt%	Ce wt%	Gd wt%	Pr wt%	Y wt%	Al wt%	Fe	Be	Cu	Ni
**Mg-Nd**	3.50 (±0.03)	2.49(±0.03)	**0.005**(±0.003)	0.05	0.01	<0.01	<0.01	0.027	0.005	<0.001	<0.001	<0.001
**Mg-Nd-Zn**	3.3(±0.1)	2.43(±0.05)	**0.77**(±0.005)	0.04	<0.01	<0.01	<0.01	0.008	61 ppm	-	<1ppm	<1ppm

Supplementary Table 2. Solubility of elements in Mg

Element	Max. solid solubility, wt.%(at%)	Eutectic temperature, °C (K)
**La**	0.23 (0.04)	612 (885)
**Nd**	3.63 (0.63)	552(825)
**Zn**	6.2 (2.4)	340 (613)

Computational Techniques. Vienna *Ab inito* Simulation Package (VASP) was used to perform quantum mechanical calculations based on density functional theory (DFT) [4]. The exchange correlation functional was described using generalized gradient approximation (GGA) using parameterization proposed by Perdew, Burke and Ernzerhof (PBE) [5]. Projector-augmented wave (PAW) pseudopotentials (PPs) with valence electron configurations [Ne]$3s^2$ and [Ar]$3d^{10}4s^2$ for Mg and Zn respectively, and frozen-core f-electrons for Nd and La was utilized in all calculations. A basis set cutoff energy of 350 eV was chosen for all calculations by carrying out convergence tests on several supercells containing Nd and Zn. Further, the k-spacing of 0.1 $Å^{-1}$ and gamma-centered k-point meshes was found to be sufficient to perform Brillouin zone integration, and achieve global energy convergence better than ~1meV. In each case, bulk structures and ones with precipitate / matrix interfaces, were relaxed till the forces on atoms were less than 0.1meV/ Å prior any further analysis. Visualization for electronic and structural analysis (VESTA) software was used to visualize the relaxed structures, and the JEMs package [6] was used to simulate diffraction patterns from the relaxed structures consisting of precipitate / matrix interfaces. MedeA software was used to generate Zn substituted γ'' precipitate structures [7].

Calculation of stacking fault and dislocation dissociation energies. To calculate stacking fault energies for various compositions we adopted the procedure previously described by Salloom et al. [8]. This involved the creation of 96 atom orthogonal cells (or 12 layers with 8 atoms per layer) with vacuum at both ends (Figure 5a). Furthermore, the cells were oriented such that $[0001]_\alpha$ was along the longer z axis, while the shearing xy plane (corresponding to $(0002)_\alpha$) was bound by two $[1\bar{1}00]_\alpha$ and $[11\bar{2}0]_\alpha$ orthogonal crystallographic directions. Faults were generated by shearing the top half of the cell along $[1\bar{1}00]_\alpha$ for ~0.28nm. This deformation creates intrinsic I2 type of stacking fault commonly seen in *hcp* structures [9,10,11]. The sheared cells were then energy minimized by allowing ionic relaxation only along the z axis. Thus, the stacking fault energy (SFE) was calculated using the equation [11],

$$SFE = (E_{fault} - E_0)/A \qquad (1),$$

where, E_{fault} and E_0 are the energies of the faulted and perfect structures respectively, and A is the interfacial area between the top and bottom slabs. In the calculations, all the solute atoms were placed in the plane of shear, and in case of the ternary Mg-Nd-Zn and Mg-La-Zn systems, the Zn atom was placed as the first nearest neighbor of Nd/La.

For activated cross slip to occur during creep, the partials must first recombine into a lattice screw dislocation, which then cross slip into a parallel slip plane [9,10,11]. Furthermore, the energy released during the recombination process will be equivalent to the dislocation dissociation energy (E_d), which can be evaluated using [11]:

$$E_d = \sigma_{r\theta}(d_{partial}\ b)\ d_{partial} \qquad (2),$$

where, $\sigma_{r\theta}$ is the stress around a screw dislocation in polar coordinates, while $d_{partial}$ and b (corresponding to $1/3\langle 1\bar{1}00\rangle_{\alpha}$) are the distance between the two partials and the Burgers vector, respectively. Equation (2) quantifies the energy required to recombine two partials separated by a distance of $d_{partial}$ by applying force $\sigma_{r\theta}(d_{partial}\ b)$ on the screw-partials. The Burgers vector was determined using the lattice parameter of Mg, while $d_{partial}$ and $\sigma_{r\theta}$ were calculated using the expressions [10]:

$$\sigma_{r\theta} = \frac{G\ b}{\pi d_{partial}} \qquad (3),$$

$$d_{partial} = \frac{G\ b}{8\ \pi\ SFE}\ \frac{2-\nu}{1-\nu}\frac{2-3\nu}{2-\nu} \qquad (4),$$

where, ν (=1/3) is the Poisson’s ratio, and SFE was obtained from DFT calculations. Thus, using equations (1)-(4) generalized stacking fault energy (GSFE) curves and dislocation dissociation energy was calculated for Pure-Mg, Mg-Nd, Mg-La, Mg-Zn, Mg-Nd-Zn and Mg-La-Zn (see Figures 5 and Supplementary Figure 9).

Supplementary Note 1

Initial Microstructure and creep response Microstructures of Mg-Nd and Mg-Nd-Zn after HPDC are presented as bright-field TEM (BFTEM) images in Supplementary Figure1. In both cases networks, like a skeletal structure, of a large intermetallic phase solidification product was observed in conjunction with the α-Mg phase. The α-Mg matrix of both alloys in the as-cast condition was free of precipitation as indicated by the absence of extra reflections in the $[0001]_{\alpha}$ selected area diffraction pattern from the α-Mg regions.

The creep response of Mg-Nd and Mg-Nd-Zn is depicted via strain versus time plots in Supplementary Figure1 and also as strain-rate versus time in Figure 1. For comparison, creep tests were also performed on polycrystalline Mg (99.9 at.% purity) at 40 and 90 MPa at 450 K (extended Supplementary Figure1). Supplementary Table 3 lists the measured creep properties of the three materials and their precipitation densities (number of particles per m^2). We find the minimum creep rate of Mg-Nd-Zn is ~20 times that Mg-Nd. The creep life times also scaled with the same order of magnitude (fourth column in Supplementary Table 3). It must be mentioned that Mg-Nd-Zn did not fail even after ~4800Hrs of creep testing, and that the test was stopped after that testing duration. Using the onset of creep (see Supplementary Table 3) from the strain vs. time plots in Fig.1a, creep lifetime of Mg, Mg-Nd and Mg-Nd-Zn was estimated (Supplementary Table 3). We found that Mg-Nd-Zn had a ~600% higher creep life than Mg-Nd. Precipitation density at the minimum creep-rate condition in Mg-Nd-Zn was also an order of magnitude greater than in Mg-Nd. However, as explained in the main text and latter in this supplementary section, precipitation strengthening alone cannot explain the spectacular creep lifetime measured in our Mg-Nd-Zn. Past studies on creep tested microstructures (specifically at minimum creep rate) of Mg-Nd-La and Mg-Nd-La-Zn did not reveal any signature of grain boundary sliding [12,13]. It is suspected that the large volume fraction of intermetallic solidification product present at the interdendritic regions restricts grain boundary sliding.

Supplementary Table 3. Summary of measured creep properties and precipitate number densities

Material	Imposed stress at 450 K (MPa)	Strain-rate at minimum creep-rate (Hr^{-1} / s^{-1})	Onset of tertiary creep (Hrs)	Time to failure (Hrs)	Precipitation density (10^{15} / m^2)
Pure-Mg	40	$5x10^{-2}$ / $5x10^{-6}$	62	87	--
Pure-Mg	90	Near instantaneous failure			
Mg-Nd	90	$1.5x10^{-3}$ / $1.5x10^{-7}$	460	727	0.9[a]
Mg-Nd-Zn	90	$3x10^{-4}$ / $3x10^{-8}$	3285	4798[c]	7.2[b]

[a] measured form combined GP and β' distribution in BFTEMs

[b] measured for γ'' distribution in BFTEMs

[c] Creep test was stopped after this duration of testing

Supplementary Note 2

Precipitation sequence in Mg-Nd and Mg-Nd-Zn alloys. This section provides a brief introduction into the precipitation sequence in Mg-Nd and Mg-Nd-Zn alloys, and the factors influencing the formation of precipitate phases.

The precipitation in Mg-Nd appear in the following sequence:
SSSS (supersaturated solid solution) → Nd-rich pockets [14-16] → GP [14-16] → β' [14-16] (*orthorhombic*, Mg_7Nd, most probable space group Cmcm [14,17], lenticular shaped – in Fig. 1b) → β_1 (*ordered-cubic*, Mg_3Nd, space group $Fm\bar{3}m$) [14-16] → β (*tetragonal*, $Mg_{12}Nd$, space group I4/mmm) [14,18] → β_e (*tetragonal*, $Mg_{41}Nd_5$, space group I4/mm) [14,18]. The β and β_e phases are observed after high temperature annealing at 500ºC [14,18].

The sequence of precipitation in Mg-Nd-Zn on the other hand is comparatively shorter:
SSSS (supersaturated solid solution) → GP zones → γ'' (*hcp*, Mg_5 (Nd,Zn), space group $P\bar{6}2m$) → γ (*cubic*, Mg_3(Nd,Zn), space group $Fm\bar{3}m$) [12,14].

Both β_1 and γ (in Mg-Nd and Mg-Nd-Zn, respectively) share a common crystal structure, possess similar and lattice parameters [12,14], and exhibit a strong tendency to form dynamically on dislocation lines (e.g. during creep reformation) instead of bulk Mg [14-16]. This behavior is due to large stress-free-transformation-strains associated with these precipitates [15,16]. Regardless, the volume fractions of β_1 and γ at low temperatures are significantly less than GP zones, β', and γ'' in their respective alloys [12]. There is also evidence that formation of β' in Mg-Nd is facilitated by applied stress and dislocations [16]. In stark contrast to these alloys, binary Mg-La does not exhibit discernable precipitation within the parent Mg lattice [19].

Supplementary Note 3

Precipitation hardening of Mg-Nd and Mg-Nd-Zn. In this section mechanical and creep strength of Mg-Nd and Mg-Nd-Zn was estimated via different models, which considers precipitate modulus, their "shearability", precipitate/matrix interfacial energies etc. [20-22].

Intermetallic precipitates typically have higher moduli than the parent matrix, and act as stiff obstacles to dislocation motion. This "modulus strengthening effect" was evaluated first by calculating the elastic constants/moduli of γ'' precipitates, which appear in very high number densities in Mg-Nd-Zn (Figure 1 and Supplementary Table 3). Our DFT calculations reveal that polycrystalline elastic moduli (G, E and B) of γ'' is similar to pure Mg (Supplementary Table 4), and the presence of γ'' precipitates will not appreciable raise the overall moduli of Mg-Nd-Zn.

Supplementary Table 4. DFT calculated elastic properties in GPa units

**Material**	**Single crystal elastic Constants**					**Hill averaged polycrystalline values**		
	**C_{11}**	**C_{12}**	**C_{13}**	**C_{44}**	**C_{33}**	**G**	**E**	**B**
γ''– $(Mg_{0.8}Zn_{0.2})_5Nd$	58	34	26	37	78	20	52	40
Pure Mg	67	19	17	16	68	21	51	34

C_{ij} are the anisotropic elastic constants, while G, E and B are the shear, Young's and Bulk moduli.

An analysis of the individual elastic constants of γ'' show that some of them are higher than Mg by a factor of two (Supplementary Table 4). Since these constants are related to shear deformation in *hcp* crystals [23], there higher values suggests (i) a possible anisotropic hardening of slip systems in Mg-matrix, and (ii) that precipitates may not be easily deformable or "cut" by the passing of dislocation. More studies are required to examine the two possibilities. Irrespective of the shearability of γ'', several theoretical frameworks have attempted to quantify the increase in the critical resolved shear strength

(CRSS) of an alloy by modeling the interaction of mobile dislocations with precipitate phases [10,20-22].

We have estimated the CRSS (Δτ) of Mg-Nd and Mg-Nd-Zn using the models proposed by Huang and Ardell using precipitate number densities (*N*) and interfacial energy (γ_i) values, and these are listed in Supplementary Table 3 [21]. Our choice was guided by two considerations: (i) *N* can be easily quantified by TEM observations (last column Supplementary Table 3) and (ii) majority of the precipitates in Mg-Nd and Mg-Nd-Zn are coherent which suggests that γ_i will have a profound influence on the strength of these alloy systems. Typically, interfacial energies of coherent interfaces range from 50-200 mJ/m^2 [24]. However, we note that due to the greater extent of electron charge localization at the γ''/Mg interface compared to β'/Mg (compare Figure 4e and Supplementary Figure 8b), the γ''/Mg interface may have lower excess values [25], at least for basal planes of Mg. Since calculation of γ_i was not in the scope of this work we have assumed similar precipitate / Mg-matrix interfacial energies in the case of Mg-Nd (β') and Mg-Nd-Zn (γ''). Using this assumption in the models listed in Supplementary Table 5, the following approximate ratio for CRSS was obtained:

$$\frac{\Delta\tau^{Mg-Nd-Zn}}{\Delta\tau^{Mg-Nd}} \approx \left(\frac{N^{Mg-Nd-Zn}}{N^{Mg-Nd}}\right)^{1/2} \qquad \textbf{(5),}$$

where, $\Delta\tau^i$ and N^i (i = Mg-Nd or Mg-Nd-Zn) are the CRSS and precipitate number densities in the two alloys. By substituting the appropriate N^i values from Supplementary Table 3 into equation (1) we find that the extraordinarily higher number density of γ'' increases the $\Delta\tau$ of Mg-Nd-Zn over that of Mg-Nd only by a factor of three (Supplementary Table 5). Note, this increase is an upper bound because in reality the interfacial energy of γ''/Mg maybe smaller than β'/Mg.

Supplementary Table 5. Models predicting increase in critical resolved shear strength (CRSS or Δτ) due to precipitation.

**Models**	**Relations for Δτ** [a]	$\frac{\boldsymbol{\Delta\tau^{Mg-Nd-Zn}}}{\boldsymbol{\Delta\tau^{Mg-Nd}}} = \left(\frac{\boldsymbol{N^{Mg-Nd-Zn}}}{\boldsymbol{N^{Mg-Nd}}}\right)^{\mathbf{1/2}}$
Shear-able "weak' precipitates	$\left(\frac{3\gamma_i b}{\Gamma}\right)^{1/2} \times N^{1/2}$	
Shear-able "strong' precipitates	$1.4\gamma_i \times N^{1/2}$	2.8 [b,c]
Non-shear-able precipitates	$\frac{Gb}{2\pi(1-\nu)} \times \frac{\ln\left(\Delta/_{\pi b}\right)}{1.43 - \sqrt{f}} \times N^{1/2}$	

[a] where, *N* is the precipitate number density per unit area, *f* volume fraction of precipitates, b Burgers vector, G shear modulus, γ_i precipitate / matrix interfacial energy, Γ dislocation line tension in Mg (assumed similar irrespective of alloying content) and ν Poisson's ratio of Mg.

[b] Δτ was calculated by assuming similar *f* (from TEM) and γ_i

Thus, our analysis based on precipitation hardening models indeed suggest an improvement in the strength of Mg-Nd-Zn due to γ'' precipitation, but these models do not provide much insight into the extraordinary creep behavior of that alloy observed in our experiments.

Supplementary Note 4

Creep response of Mg-Nd and Mg-Nd-Zn via dislocation climb. Several studies have indicated that the creep response of Mg alloys is a diffusion dominated phenomenon, which is manifested as either vacancy mediated dislocation climb, the viscous drag of dislocation by solutes or a combination of both [26,27]. The role of activated cross-slip have been discussed on the main manuscript and in a previous section. Therefore, this section will evaluate the creep response of Mg-Nd and Mg-Nd-Zn under both mechanisms using the Bird, Mukherjee and Dorn (BMD) equation for steady state creep [28]:

$$\frac{\dot{\varepsilon}kT}{DGb} = A\left(\frac{\sigma}{G}\right)^{n} \qquad \textbf{(6)}$$

where, $\dot{\varepsilon}$ is the steady state strain rate, D is the diffusivity, G the shear modulus, b the Burgers vector, σ the stress, k is the Boltzmann's constant, T is the temperature and A is a constant. Furthermore, the stress exponent "*n*" in equation (6) depends on the operative mechanism (n = 4-7 for dislocation climb or n=3 viscous drag). For the present calculations we have used G = 20 GPa from Supplementary Table 4, T = 450 K, $b \approx 10^{-10}$ m and σ = 90 MPa.

The critical parameter in equation (6) is the diffusivity D, given by the Arrhenius equation $D = Do\ exp(-\frac{Q}{kT})$. The activation energy $Q = E_v^f + E_v^m$, where E_v^f and E_v^m are the vacancy formation and migration energy respectively. By substituting these values in equation (6) we have

$$\dot{\varepsilon} = A' \ kTG\left(\frac{\sigma}{G}\right)^{n} e^{\frac{-(E_v^f+E_v^m)}{kT}} \qquad \textbf{(7)}$$

The new constant A' incorporates the exponential prefactor *Do*. We have modeled *D* on the basis of Mg vacancy diffusion or substitution/self-diffusion in Mg, Mg-Nd and Mg-Nd-Zn by considering diffusion between two $(0002)_\alpha$ planes (out-of-plane or *oop*) and within a $(0002)_\alpha$ plane (in-plane or *ip*) [26,27,29]. Our DFT calculated diffusivity values

are listed in Supplementary Table 6, and shows that the *oop* diffusivity of Mg-Nd-Zn is significantly lower (by two orders of magnitude) than that for Mg-Nd. Although we have only considered Mg vacancy diffusion, we expect Nd diffusion will also be high in the presence of Zn because Nd bonds very well with Zn (Fig.4c). This is based on our charge density calculations in Mg-Nd-Zn (Figure 4c in main text), which indicated high localization of electron density around Nd and Zn. Notwithstanding, our DFT calculations indicate that *oop* diffusivities will be strongly influence the creep rates. To establish this, we first estimated *A'* in equation (7) with pure Mg diffusivity values (Supplementary Table 6) and creep rate at 40 MPa (Supplementary Table 3), and then used this constant to calculate the creep-rates of Mg-Nd and Mg-Nd-Zn for n=3 and n=5 (mid-way between 4 and 7). Supplementary Table 7 list the results from our calculations. Whilst it is difficult to conclusively comment on the dominant mechanisms from these results, it appears that the observed strain rates in Supplementary Table 3 are somewhere in between *oop* dislocation climb (n=5) and viscous glide (n=3) in Supplementary Table 7. Regardless, we have unambiguously established that creep rate estimated via the BMD model for Mg-Nd-Zn is less than that in Mg-Nd by at least an order of magnitude, which is in excellent agreement with our experimental observations.

Supplementary Note 5

Role of La on creep deformation. Past studies have indicated that La solutes improve castability, in comparison to Mg-Nd and Mg-Ce, by reducing hot tearing in the as-solidified product [1,30]. However, high-pressure die-cast (HPDC) Mg-La had the worst creep response of the three alloys [15], which has been attributed to the low solid solubility of La in the Mg matrix [15] (also see Supplementary Table 2). Microstructure of HPDC Mg-La comprises of Mg matrix surrounded by a large volume fraction interdendritic solidification phase skeleton [1,15]. Atom probe studies show that La preferentially partitions in such interdendritic phases in HPDC Mg-Nd-Zn alloys [12]. The same work also indicated very small composition of La (<0.01 at%) inside the Mg matrix [12]. Therefore, a possibility that La may influence the creep behavior mandated an assessment of La's influence in the present study.

The role La was examined by calculating the minimum energy paths for vacancy diffusion (Supplementary Figure6) and GSFE curves of binary Mg-La and Mg-La-Zn alloys (Supplementary Figure9). The computed results indicated that vacancy migration barrier energies follow the trend: $E^{oop}_{Mg-La-Zn} > E^{oop}_{Mg-La} > E^{oop}_{Mg}$ (Supplementary Figure6a), and $E^{ip}_{Mg-La-Zn} \approx E^{ip}_{Mg} > E^{ip}_{Mg-La}$ (Supplementary Figure 6b), which is similar to that seen in Mg-Nd and Mg-Nd-Zn alloys. Thus, out of plane vacancy diffusion (for dislocation climb) will be prevented when Zn is present near La atoms. Like Mg-Nd and Mg-Nd-Zn alloys, the SFEs in Mg-La and Mg-La-Zn alloys vary as $I2_{Mg\text{-}La\text{-}Zn} < I2_{Mg\text{-}La} < I2_{Pure\text{-}Mg}$ (Supplementary Figure 9a). Furthermore, Zn addition substantially increases the dislocation dissociation energy (Supplementary Figure 9b); and hinder activated cross-slip. Taken together we find the addition of Zn to Mg-rare earth alloys (or at least Nd and La) improves creep resistance by limiting dislocation climb and activated cross-slip. However, the beneficial effects resulting from Zn-La interaction (similar to Zn-Nd interactions) will depend on the La content in the Mg matrix.

Supplementary Note 6

Summary. The remarkable creep strength of the Zn containing was examined with well-known models. The cast Mg-Nd (Mg-0.6Nd-0.4La in at%) and Mg-Nd-Zn (Mg-0.6Nd-0.4La-0.3Zn in at%) alloys consisted of precipitation free Mg-matrix (insets Supplementary Figure1), and past work has suggested that La has very low solubility in Mg (Supplementary Table 2) and primarily resides within the interdendritic product. Both alloys experience dynamic precipitation when exposed to a constant load stress of 90 MPa at 450 K. The precipitation in Mg-Nd-Zn contained significantly higher number density of fine scale precipitates than Mg-Nd (Supplementary Table 1). While precipitates in primarily formed on the basal planes in Mg-Nd-Zn, they formed on prismatic planes in Mg-Nd (Fig.1). Our DFT calculations suggest that the presence of Zn (near Nd) localizes the electron charge density along the *c*-axis of *hcp*-Mg in Mg-Nd-Zn alloy, and likely drives γ'' nucleation on basal planes. The dispersion/number density of precipitates may be proportional to the elemental distribution of Zn in Mg-Nd-Zn. The Huang and Ardell model indicated that this high number density of γ'' increases the yield strength of Mg-Nd-Zn only by a factor of three over Mg-Nd (Supplementary Table 5), which is in excess of ~100MPa for typical Mg-RE alloys [31]. Our results reveal that plastic deformation occurs at 90 MPa in both systems, which means that deformation mechanisms other than basal/prismatic/pyramidal slip is contributing to the overall plastic strain.

Based on past work and our present results we show that vacancy diffusion assisted dislocation climb is a probable contributing factor. However, the enhanced covalent bonding due to charge localization near the solutes and γ'' in Mg-Nd-Zn creates stronger Mg-Mg bonds (Fig.4). Consequently, this increases the vacancy migration barriers and diffusivities at 450 K (Supplementary Table 6). Such high energetic penalty for Mg vacancy migration contributes to reduction in the observed strain rates and creep lifetime of Mg-Nd-Zn (Fig.1a and Supplementary Figure 1) by an order of magnitude over Mg-Nd. Finally, reduction in the stacking fault and dislocation dissociation energies (Fig 5 and Supplementary Figure 9) obtained after Zn addition will further contributed to the overall creep resistance.

Supplementary Table 6. DFT calculated diffusion energetics and diffusivity values.

Material	Vacancy formation energy (eV)	Out-of-plane diffusion path			In-plane diffusion path		
		Barrier (eV)	Activation energy (eV)	Diffusivity at 450 K (m^2/s)	Barrier (eV)	Activation energy (eV)	Diffusivity at 450 K (m^2/s)
Pure-Mg	0.88	0.39	1.27	1.3×10^{-20}	0.39	1.27	1.8×10^{-20}
Mg-Nd	0.77	0.75	1.52	1.9×10^{-23}	0.30	1.07	3.0×10^{-18}
Mg-Nd-Zn	0.79	0.89	1.86	3.3×10^{-25}	0.36	1.15	3.5×10^{-19}

Supplementary Table 7. Calculated creep rates (in sec^{-1} units) for Mg-Nd and Mg-Nd-Zn for dislocation climb (n=5) and viscous drag (n=3).

Material	Out-of-plane (*oop*)		In-plane (*ip*)	
	n = 3	n = 5	n = 3	n=5
Mg-Nd	3.91×10^{-4}	7.46×10^{-9}	8.01×10^{-4}	8.01×10^{-4}
Mg-Nd-Zn	6.79×10^{-6}	1.30×10^{-10}	9.27×10^{-5}	9.50×10^{-5}

Supplementary References

1. Easton, M., Gavras, S., Gibson, M., Zhu, S., Nie, J. F., & Abbott, T., Hot tearing in magnesium-rare Earth alloys. In Magnesium Technology 2016 (pp. 123-128). Springer International Publishing, *and references there in*
2. Cerezo, A & Davin, L., Aspects of the observation of clusters in the 3-dimensional atom probe, Surf. Interface. Anal. 39, 184-188 (2007)
3. Hyde, J.M., E.A. Marquis, Wilford, K.B. & Williams, T.J., A sensitivity analysis of the maximum separation method for characterization of solute clusters, Ultramicroscopy, 111, 440-447 (2011)
4. Kresse, G. & Furthmuller, J., Efficiency of ab-initio total energy calculations for metals and semiconductors using plane-wave basis set, *Comp. Mater. Sci*, 6, 15-50 (1996)
5. Perdew, J.P., Burke, K. & Ernzerhof, M., Generalized gradient approximation made simple, *Phy. Rev. Lett*, 77, 3865-3868 (1996)
6. Stadlemann, P.A., JEMS – EMS java version, 2004
7. http://www.materialsdesign.com/software
8. Salloom, R., Banerjee, R., & Srinivasan, S. G, Effect of β-stabilizer elements on stacking faults energies and ductility of α-titanium using first-principles calculations. Journal of Applied Physics, 120, . (2016)175105.
9. Hull, D. & Bacon, D.J., Introduction to Dislocations, Butterworth-Heinmann, 4th ed, USA,
10. Anderson, P. M., Hirth, J. P., & Lothe, J. (2017). Theory of Dislocations. Cambridge University Press.
11. Rasmussen, T., Jacobsen, K. W., Leffers, T., Pedersen, O. B., Srinivasan, S. G., & Jonsson, H., Atomistic determination of cross-slip pathway and energetics. Physical Review Letters, 79(19),(1997) 3676.
12. Choudhuri, D., Jaeger, D., Gibson, M.A., & Banerjee, R., Role of Zn in enhancing the creep resistance of Mg-RE alloys, Scr. Mater., 86, 32-35 (2014)
13. Choudhuri, D., Jaeger, D. L., Srivilliputhur, S., Gibson, M. A., & Banerjee, R. (2015). Creep response of a Zn containing Mg-Nd-La alloy. In Magnesium Technology 2015 (pp. 35-39). Springer International Publishing.
14. Easton M.A., Gibson M.A., Qiu D., Zhu S,-M., Grobner J., Schmid-Fetzer R., Nie J.-F., Zhang M.X. The role of crystallography and thermodynamics on phase selection in binary magnesium-rare earth (Ce of Nd) alloys, Acta Mater 60: 4420-4430(2012)
15. Zhu, S.M., Gibson, M.A., Easton, M.A. & Nie, J.F., The relationship between microstructure and creep resistance in die-cast magnesium-rare earth alloys, Scr. Mater, 63, 698-703 (2010)
16. Nie, J.-F., Precipitation and Hardening in magnesium alloys, Metall. Trans. A, 43A (1985)3891 - 3939

17. Choudhuri, D., Dendge, N., Nag, Meher, S., Alam, T., Gibson, M.A. & Banerjee, R., Homogeneous and heterogeneous precipitation mechanisms in a binary Mg-Nd alloy, *J. Mater. Sci.*, 49, 6986-7003 (2014)
18. Choudhuri, D., Dendge, N., Nag, S., Gibson, M.A. & Banerjee, R., Role of applied uniaxial stress during the creep testing on precipitation in Mg-Nd alloys, Mater. Sci. Eng. A., 612, 140-152 (2014)
19. Banerjee, S. & Mukhopadhayay, P., Phase Transformations: Examples from Titanium and Zirconium Alloys, Pergamon Materials Series, Ed. R.W. Cahn, GB
20. Dieter, G. E., & Bacon, D. J. (1986). Mechanical metallurgy (Vol. 3). New York: McGraw-Hill.
21. Huang, J. C., & Ardell, A. J. (1987). Strengthening mechanisms associated with 1 particles in two Al-Li-Cu alloys. *Le Journal de Physique Colloques*,*48*(C3), C3-373.
22. Nie, J. F., Precipitation and Hardening in magnesium alloys, Metall. Trans. A, 43A (1985) 3891 – 3939
23. Nye, J. F. (1985). *Physical properties of crystals: their representation by tensors and matrices*. Oxford university press.
24. Porter, D. A., Easterling, K. E., & Sherif, M. (2009). *Phase Transformations in Metals and Alloys, (Revised Reprint)*. CRC press.
25. Choudhuri, D., Banerjee, R., & Srinivasan, S. G., Interfacial structures and energetics of the strengthening precipitate phase in creep-resistant Mg-Nd-based alloys. Scientific reports, 7, (2017) 40540.
26. Vagarli, S.S. & Langdon, T.G., Deformation mechanism in H.C.P. metals at elevated temperatures – I. Creep behavior of magnesium, *Acta Metall.*, 29 ,1969-1982 (1981)
27. Vagarli, S.S. & Langdon, T.G., Deformation mechanism in H.C.P. metals at elevated temperatures – II. Creep behavior of Mg-0.8%Al solid solution alloy, *Acta Metall.*, 30, 1157-1170 (1982)
28. Murty, K. L., Mohamed, F. A., & Dorn, J. E. (1972). Viscous glide, dislocation climb and Newtonian viscous deformation mechanisms of high temperature creep in Al-3Mg. *Acta Metallurgica*, *20*(8), 1009-1018.
29. Shewmon, P. (1989). Diffusion in solids. *The Minerals, Metals & Materials Society, Diffusion in Solids. Second Edition. (Retroactive Coverage)(United States), 1989*, 246.
30. Easton, M. A., Gibson, M. A., Zhu, S., & Abbott, T. B., An a priori hot-tearing indicator applied to die-cast magnesium-rare earth alloys. Metallurgical and Materials Transactions A, 45(8), (2014)3586-3595.
31. Joost, W. J., & Krajewski, P. E., Towards magnesium alloys for high-volume automotive applications. Scripta Materialia, 128, (2017)107-112.
